# Genetic diversity and population structure of *Echinococcus multilocularis*: An *in-silico* global analysis

**DOI:** 10.5455/javar.2024.k772

**Published:** 2024-06-04

**Authors:** Ayed Alshammari, Muhammad Irshad Subhani, Majed H. Wakid, Abdulsalam A.M. Alkhaldi, Shujaat Hussain, Muhammad Abdullah Malik, Muhammad Saqib, Warda Qamar, Mughees Aizaz Alvi

**Affiliations:** 1 Department of Biology, College of Science, University of Hafr Al Batin, Hafr Al Batin, Saudi Arabia; 2 Department of Clinical Medicine and Surgery, University of Agriculture, Faisalabad, Pakistan; 3 Department of Medical Laboratory Sciences, Faculty of Applied Medical Sciences, King Abdulaziz University, Jeddah, Saudi Arabia; 4 Special Infectious Agents Unit, King Fahd Medical Research Center, Jeddah, Saudi Arabia; 5 Biology Department, College of Science, Jouf University, Sakaka, Saudi Arabia; 6 Faculty of Veterinary and Animal Sciences, PMAS Arid Agriculture University, Rawalpindi, Pakistan; 7 Department of Parasitology, University of Agriculture, Faisalabad, Pakistan; † Authors contributed equally to this work.

**Keywords:** *Echinococcus multilocularis*, *cox*1, *nad*1, genetic variability

## Abstract

**Objective:**

Alveolar echinococcosis is caused by *Echinococcus multilocularis*, a parasite of zoonotic significance with a wide range of intermediate and final hosts, and the parasite survives successfully in diversified conditions. Plentiful studies have been done to study the genetic structure of the population of the parasite and the level of intimate kinship using mitochondrial (mt) DNA. The present study was conducted to investigate the population structure, genetic variation, and phylogenetic relationship of various isolates of *E. multiocularis* submitted to GenBank worldwide. Sequences of mt genes (mt-cytochrome c oxidase (*cox*1), mt-NADH dehydrogenase* (nad*1)) of *E.* *multilocularis* were analyzed to achieve the set goals.

**Materials and Methods:**

A total of 275 and 124 gene sequences of mt-*cox*1 and mt-*nad*1 belonging to *E*. *multilocularis,* respectively, were retrieved from the National Center for Biotechnology Information GenBank. The retrieved sequences were subjected to alignment with respective reference sequences using MEGA software. The PopArt software was used to establish median-joining networks, while DnaSp was used to calculate neutrality and diversity indices. MrBayes software was used to investigate the phylogenetic association between haplotypes based on Bayesian phylogeny.

**Results:**

Approximately 13 and 20 distinctive haplotypes of *nad*1 and *cox*1 genes, respectively, were observed in the present study. In both of the mt genes, diversity indices indicated low haplotype (mt-*cox*1 = 0.140; mt-*nad*1 = 0.374) and nucleotide (mt-*cox*1 = 0.00111; mt-*nad*1 = 0.00287) diversities. The values of Tajima’s D and Fu Fs for a population of both of the genes under study were found to be negative.

**Conclusion:**

This study is a maiden attempt to provide insights into the population structure and genetic variation of *E. multilocularis* on a global scale. However, it is suggested that to better understand the population structure and genetic diversity of *E*.* multilocularis,* more geographical locations and amplifications of full-length gene sequences should be considered, which could be helpful in widening the insights into the genetic diversity of *E*. *multilocularis*.

## Introduction

Parasitic infections causing infectious diseases are gaining significant importance nowadays in both the veterinary and public health sectors, resulting in both economic losses and serious illness [1–4]. Alveolar echinococcosis (AE), which is thought to be among the most dangerous zoonotic diseases in nontropical areas, is caused by a form of parasitic tapeworm called *Echinococcus multilocularis* [5], which occurs predominantly in the northern half of the globe and can cause hyperplasia, fibrosis, putrefaction of liver tissues, and hepatic fibrosis [6]. Human AE disease is brought about by the accidental ingestion of food sullied with eggs, which form microcystic metacestode vesicles in the liver [7].

Chiefly, *E. multilocularis* maintains a sylvatic life cycle in which small mammals, i.e., arvicoline rodents, serve as intermediate hosts and wild canids, i.e., foxes (*Vulpes* *vulpes*) and coyotes (*Canis* *latrans*), as final hosts. However, a synanthropic cycle aided by domestic carnivores, i.e., dogs, could be expected, which can be quite important in some regions for the spread of the parasite [8]. *Echinococcus multilocularis* adult forms could be found in the small intestine of the final hosts, ranging in size from 1.2 to 4.5 mm [9]. The proglottids are secreted in the environment through the feces of specific hosts, which contain embryonated eggs. The eggs are distinguished by a thick and extremely tough keratinized embryophore layer, which enables them to endure in the environment for a long time while being vulnerable to desiccation and higher temperatures [10]. The eggs are ingested by intermediate hosts. Upon ingestion, the oncosphere (larvae) is released from the eggs. The larvae pierce the intestine to enter the bloodstream and reach the liver (the primary target). In the liver, the metacestodal stage of the parasite grows and multiplies asexually, resulting in the production of protoscolices. These protoscolices get themselves attached to the intestinal wall of the intermediate hosts, where they convert into adult worms. Finally, the intermediate host is consumed by the final host, starting a new life cycle [11].

The geographic distribution and prevalence of the parasite in wild hosts are expanding worldwide because of anthropogenic activities [12]. *Echinococcus multilocularis* has been reported to cycle throughout many cities in Europe [13] and Japan over the past ten years, following the colonization of urban areas by red fox populations [14]. In addition, the recent growth of coyote populations in North America and the high prevalence of the parasite in coyote populations in urban areas [15] may pose a high risk of exposure to humans, as has been seen, for instance, in the extensively populated prairies of the Tibetan plateau and China, where the rise in the population of definitive hosts led to a high prevalence [16].

Frequent examination of mitochondrial (mt) DNA sequences was done to determine the population structure and degree of close kinship due to their high mutation rates and maternal inheritance [17]. Because of their vast variety of hosts and ecological diversification, molecular studies can play a critical role in determining the distribution of *E. multilocularis*. The diversity indices indicate that forecasting antigenic variation and phylogenetic association could be better understood by employing the main population in defined habitats. Moreover, numerous investigations have been done to identify the genetic variations in various strains of *E. multiloclaris* through the use of partial sequences of mt-cytochrome c oxidase (*cox*1) and mt- NADH dehydrogenase* (nad*1) mt genes. Therefore, to better understand the population dynamics of *E.* *multilocularis*, it is vital to investigate the genetic diversity of *E. multilocularis* isolates on a global scale. Furthermore, there is very little data available regarding the epidemiology and population structure of *E. multilocularis*. To address the identified research gap, the existing study aimed to examine the population structure, genomic variation, and phylogenetic association of *E. multilocularis* using mt gene sequences of mt-*cox*1 and mt-*nad*1 submitted to the National Center for Biotechnology Information database from various geographical locations around the world.

## Materials and Methods

### Collection of data

A dataset of 399 gene sequences was created after filtering the obtained mt-*cox*1 (*n *= 275) and mt-*nad*1 (*n* = 124) gene sequences of *E. multilocularis* submitted to the NCBI database by February 10, 2023.

### Alignment and phylogenetic analysis

The MEGA software version 11 was used to import the FASTA format of all the gene sequences [18]. First, all the sequences were subjected to cutting from both ends by using reference sequences of both the genes mt-*cox*1 (accession no. MZ026358) and mt-*nad*1 (accession no. AB018440). A total of 399 gene sequences, i.e., 497 bp mt-*cox*1 = 275 and 285 bp mt-*nad*1* = *124, were subjected to bioinformatic analyses after the elimination of short gene sequences upon filtration. The phylogenetic association between different haplotypes of *cox*1 and *nad*1 was inferred through the application of the Bayesian approach and MrBayes v.3.1.1 software [19]. The parameters were lodged in every 1,000 states using a length of 5,000,000 states. 25% of the data was deleted as a “burn-in.” The posterior distribution of the parameters was evaluated using the MCMC sampling method.

### Analyses of haplotypes and networking

Haplotype analyses were done through the examination of sequences in FASTA format using the DnaSP tool [20]. Neutrality indices, numbers of haplotypes and nucleotides, and nucleotide and haplotype change values were used to determine the genetic makeup of both genes. After the conversion of sequences to Nexus format [22], a haplotype network was created using PopArt [21].

## Results

A total of 399 gene sequences from *E. multilocularis* were examined during the present study. The gene isolates of *E. multilocularis* were obtained from the NCBI database. A total of 275 gene sequences of mt-*cox*1 gene and 124 of mt-*nad*1 gene were obtained from 13 countries (Table 1).

**Table 1.** Accession number of mt-*cox*1 and mt-*nad*1 gene fragments of *E*. *multilocularis* isolates used in the study.

mt-*cox*1			mt-*nad*1		
Origin	No. of isolates	Accession numbers	Origin	No. of isolates	Accession numbers
Japan	1	AB385610	Japan	2	AB018440, NC000928
Russia	6	AB688128-29/32, AB777915/17/19	Iran	32	AB617846-47-48/50-51-52-53-54-55, AB621793-94-95-96-97-98-99-00-01, KX186699-KX186700-01-02/04-05, AB720065-66-67-68-69, KT318129-30, KT033489
Poland	30	KY205679-80-81-82-83/85/87-88-89-90-91, MW255900-01-02/04/06/07-08/10-11/13-14-15/92-93-94-95-96-97, MN444798	Poland	12	AJ132907-08-09-10, MH986749-50-51, JX266825-26, MN444804-05, AJ237639

mt-CO1			mt-ND1		
Origin	No. of isolates	Accession numbers	Origin	No. of isolates	Accession numbers
Mongolia	1	KC893696	Sweden	4	KX384668-69-70-71
China	193	AB461417, AB477010-11-12, AB491457-58-59-60-61, JF906152-53, MN251846-47/49, MH259764-65-66-67-68-69-70/72/74, KY446474-75-76-77-78-79-80-81-82-83-84-85-86-87-88-89-90-91-92-93-94-95-96-97-98-99, KY446501-02-03-04-05-06-07, KY354083-84-85-86-87-88-89-90-91-92-93-94-95, KY328672-73-74-75-76-77-78-79-80-81-82-83-84-85-86-87-88-89-90-91-92-93/94/96-97, KY062624-25-26-27-28-29-30-31-32-33-34, MK598850, KX685923-24-25-26, MZ026301-02-03-04-05-06-07-08-09-10-11-12-13-14-15-16-17-18-19-20-21-22-23-24-25-26-27-28-29-30-31-32-33-34-35-36-37-38-39-40-41-42-43-44-45-46-47-48-49-50-51-52-53-54-55-56-57-58-59-60-61-62-63-64, KT965438-39-40-41-42, MH211144-45-46-47-48/50-51-52-53/55-56-57-58-59	China	12	KY094609, EU704122-23-24, KU723572, AY389984, MH259775-76-77-78, MN448476/78,
France	1	AB461413	Turkey	6	MK248696, MK248702-03-04-05-06
Slovakia	19	AB461414, OP225830, OP225946-47-48-49-50-51-52-53-54, OP225398, OP225402/45/48/55, OP225644, MN444797-98	Slovakia	13	MW326786-87, MN444801-02-03, MW343787-88-89, MW357715, MW366778-79, MW384819-20
USA	4	OK330092, AB461418, LC380931, NC000928	Germany	2	AJ237640, AB668376
Austria	1	AB461412	Austria	2	MN251882, MN444806
Hungary	1	MN444795	Hungary	2	MN444799, MN444800
Switzerland	2	MT461410-11	Switzerland	1	KR870967
Kyrgyzstan	4	MN829532/36-37-38	Estonia	1	AY855918
			Slovenia	1	MW560731
			Kazakhstan	1	OM640356

### Analyses of polymorphism and haplotypes

Distinct mutations were found in both genes. Approximately 24 distinct mutations were present in mt-*cox*1 gene, and 14 distinct mutations were present in mt-*nad*1 gene. After analyses of 275 gene sequences of mt-*cox*1 gene, 20 distinct haplotypes were identified (Table 2). Out of 275 gene sequences, 175 were associated with a single haplotype, the Hap01, which acts as the dominant haplotype. Whereas, upon analysis of 124 mt-*nad*1 gene sequences, 13 distinct haplotypes were identified (Table 3). Out of 97 gene sequences, 32 were present as a single haplotype, with Hap01 acting as the dominant haplotype among these.

### Haplotype network

**Figure 1.**
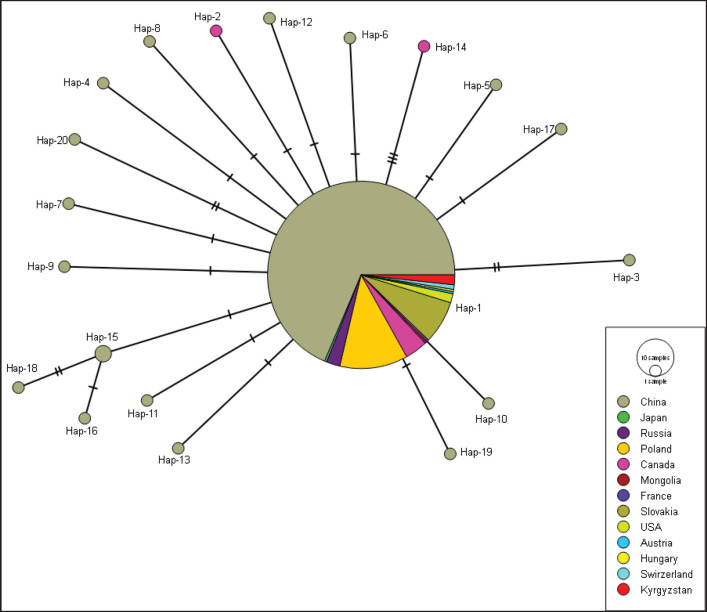
Appearance of mt-*cox*1 (497 bp) haplotypes *E*.* multilocularis* sequences.

In mt-*cox*1 gene network, 20 different haplotypes were present (Table 2). As per the analyses, the difference between the primary haplotype and the other haplotypes was 1–3 mutations. Hap01 was identified as one of the most prevalent haplotypes, which accounted for 92.72% (255/275) of the total. Hap15 came in second with 0.72% (2/275). 90% (18/20) of the network’s haplotypes were all distinct single haplotypes. Canada (*n = *2) and China (*n* = 16) each contributed a single haplotype (Fig. 1).

**Figure 2.**
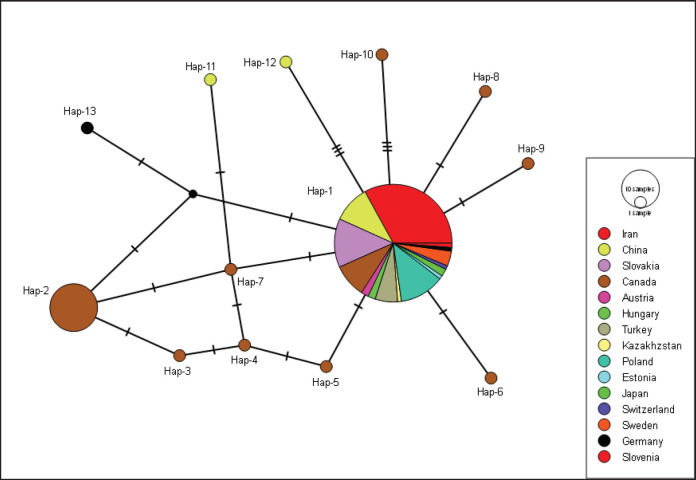
Appearance of mt-*nad*1 (285 bp) haplotypes *E*. *multilocularis* sequences.

In mt-*nad*1 gene network, there were 13 haplotypes (Table 3). The difference between the primary haplotypes and the other haplotypes was 1–4 in this network. The most prevalent haplotype, Hap01, made up 78.22% (97/124) of the haplotype network, whereas Hap02 made up 12.90% (16/124). 84.61% (11/13) of the haplotype network was made up of a single distinct haplotype. Germany (*n = *1), China (*n = *2), and Canada (*n = *8) each had a single haplotype (Fig. 2).

**Table 2.** Haplotypes of mt-*cox*1 sequences of *E*. *multilocularis* and accession numbers of isolates forming groups.

Name of haplotype	No. of Isolates	Accession number	Name of haplotype	No. of Isolates	Accession number
Hap01	255	OK330092-USA, AB461418-USA, AB461417-China, KY205687-Poland, KY205688-Poland, KY205689-Poland, KY205690-Poland, KY205691-Poland, KY205679-Poland, KY205680-Poland, KY205681-Poland, KY205682-Poland, KY205683-Poland, KY205685-Poland, AB461414-Slovakia, AB688128-Russia, AB688129-Russia, AB688132-Russia, AB777915-Russia, AB777917-Russia, AB777919-Russia, AB461412-Austria, AB461413-France, AB477010-China,	Hap02	1	KC550004-Canada
		AB477011-China, AB477012-China, MN251846-China, MN251847-China, MN251849-China, MW255892-Poland, MW255893-Poland, MW255894-Poland, MH259764-China, MN829532-Kyrgyzstan, MN829536-Kyrgyzstan, MN829537-Kyrgyzstan, MN829538-Kyrgystan, MW255900-Poland, MW255901-Poland, MW255902-Poland, MW255904-Poland, MW255906-Poland, MW255907-Poland, MW255908-Poland, MW255910-Poland, MW255911-Poland, MW255913-Poland, MW255914-Poland, MW255915-Poland,	Hap03	1	MH259772-China
		MK843308-Canada, MK843309-Canada, MT461409-Canada, MT461410-Switzerland, MT461411-Switzerland, MW255895-Poland, MW255896-Poland, MW255897-Poland, KC550007-Canada, MH259765-China, MH259766-China, MH259767-China, MH259768-China, MH259769-China, MH259770-China, MH259774-China, AB385610-Japan, LC380931-USA, NC000928-USA, KY446479-China, KY446490-China,	Hap04	1	KY446478-China
		KY446501-China, KY446502-China, KY354084-China, KY446475-China, KY446476-China, KY446482-China, KY446487-China, KY446491-China, KY354083-China, KY354085-China, KY446474-China, KY446477-China, KY446483-China, KY446484-China, KY446485-China, KY446492-China, KY446493-China, KY446494-China, KY446495-China, KY446496-China, KY446497-China, KY446499-China, KY446504-China,	Hap05	1	KY446480-China
		KY446505-China, KY446506-China, KY354087-China, KY328674-China, KY328694-China, KC550001-Canada, KY354093-China, KY328673-China, KY328675-China, KY328678-China, KY328679-China, KY328685-China, KY328689-China, KY328672-China, KY328683-China, KY328686-China, KY328691-China, KY328692-China, KY328693-China, KY328680-China, KY328684-China, KY354088-China, KY328690-China,	Hap06	1	KY446481-China
		KY354094-China, KY062624-China, KY354092-China, KY062633-China, KY062634-China, KY062628-China, KY062632-China, MK598850-China, KY062625-China, KY062631-China, KY354095-China, KY354090-China, KY328676-China, KY328687-China, KY328688-China, KX685923-China, KX685924-China, KX685925-China, KX685926-China, AB491457-China, AB491458-China, AB491459-China, AB491460-China,	Hap07	1	KY446503-China
		AB491461-China, JF906152-China, JF906153-China, KC582621-Canada, KC582623-Canada, KC582624-Canada, KC582625-Canada, KC582627-Canada, OP225830-Solvakia, OP225948-Solvakia, OP225949-Solvakia, OP225950-Solvakia, OP225951-Solvakia, OP225952-Solvakia, OP225953-Solvakia, OP225954-Solvakia, OP225398-Solvakia, OP225402-Solvakia, OP225448-Solvakia, OP225555-Solvakia, OP225945-Solvakia, OP225946-Solvakia, OP225947-Solvakia, MN444795-Hungary,	Hap08	1	KY354086-China
		MN444796-Solvakia, MN444797-Solvakia, MN444798-Poland, KY062627-China, KY354091-China, KY328681-China, KC893696-Mongolia, KY062629-China, KY354096-China, KY354097-China, KY062626-China, MZ026309-China, MZ026319-China, MZ026320-China, MZ026321-China, MZ026322-China, MZ026323-China, MZ026324-China, MZ026325-China, MZ026326-China, MZ026327-China, MZ026328-China, MZ026329-China, MZ026330-China,	Hap09	1	KY446486-China
		MZ026331-China, MZ026332-China, MZ026333-China, MZ026334-China, MZ026335-China, MZ026336-China, MZ026337-China, MZ026338-China, MZ026339-China, MZ026340-China, MZ026341-China, MZ026342-China, MZ026343-China, MZ026344-China, MZ026345-China, MZ026346-China, MZ026347-China, MZ026348-China, MZ026349-China, MZ026350-China, MZ026351-China,	Hap10	1	KY446488-China
		MZ026352-China, MZ026353-China, MZ026363-China, OP225644-Slovakia, MZ026301-China, MZ026302-China, MZ026303-China, MZ026304-China, MZ026305-China, MZ026306-China, MZ026307-China, MZ026308-China, MZ026310-China, MZ026311-China, MZ026312-China, MZ026313-China, MZ026314-China, MZ026315-China, MZ026316-China, MZ026317-China, MZ026318-China, MZ026354-China, MZ026355-China, MZ026356-China,	Hap11	1	KY446489-China
		MZ026357-China, MZ026358-China, MZ026359-China, MZ026360-China, MZ026361-China, MZ026362-China, MZ026364-China, KY328677-China, KY328682-China, KY354089-China, KY062630-China, KT965438-China, KT965440-China, KT965439-China, KT965441-China, KT965442-China, MH211144-China, MH211145-China, MH211147-China, MH211148-China, MH211150-China, MH211151-China, MH211152-China	Hap12	1	KY446498-China
			Hap13	1	KY446507-China
			Hap14	1	KC582626-Canada
			Hap15	2	MH211146-China,MH211158-China
			Hap16	1	MH211153-China
			Hap17	1	MH211155-China
			Hap18	1	MH211156-China
			Hap19	1	MH211157-China
			Hap20	1	MH211159-China

**Figure 3.**
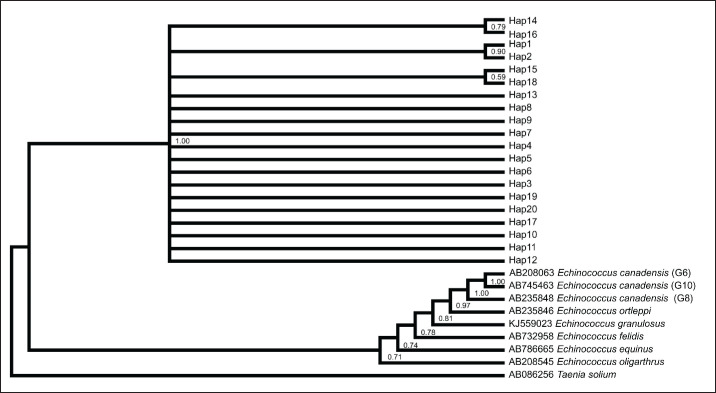
Phylogenetic tree view of *E*. *multilocularis* sequences using mt-*cox*1 (497 bp).

**Figure 4.**
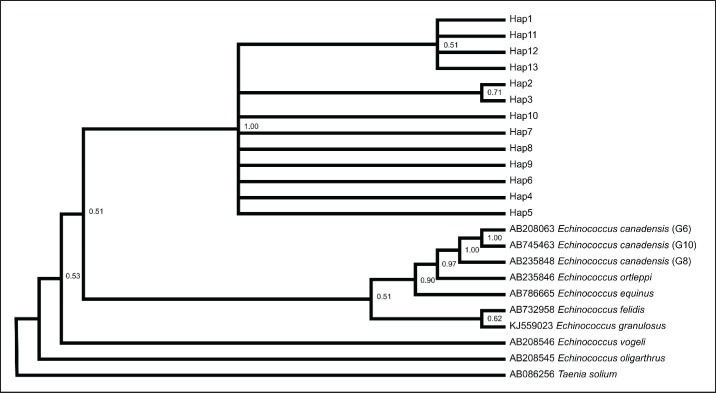
Phylogenetic tree view of *E*. *multilocularis* sequences using mt-*nad*1 (285 bp).

The findings of the haplotype network and phylogenetic analyses were consistent with each other. In the construction of both phylogenetic trees, *Taenia solium* was added as an outgroup. After the construction of the tree, sister relationships have been found between* Elodea. canadensis *and* Echinococcus ortleppi *while* Echinococcus oligarthra *occupies the basal side of the tree. The tree was generated after the alignment of the gene sequences of mt-*cox*1 and mt-*nad*1 gene sequences, as shown in Figures 3 and 4, respectively. The haplotypes, i.e., Hap03, Hap16, and Hap18 of mt-*cox*1 gene were found farther apart with mutations at three different points, while, in the case of mt-*nad*1 gene, only Hap02 was found farther apart, having mutations at four points.

**Table 3.** Haplotypes of mt-*nad*1 sequences of *E*. *multilocularis* and accession numbers of isolates forming groups.

Name haplotype	No. of isolates	Accession numbers
Hap01	97	AB018440-Japan, NC000928-Japan, AB617846-Iran, AB617847-Iran, AB617848-Iran, AB617850-Iran, AB617851-Iran, AB617852-Iran, AB617853-Iran, AB617854-Iran, AB617855-Iran, AB621793-Iran, AB621794-Iran, AB621795-Iran, AB621796-Iran, KX186700-Iran, AB621797-Iran, AB621798-Iran, AB621799-Iran, KX186701-Iran, KX186702-Iran, AB621800-Iran, AB720065-Iran, AB62180-Iran, AB720067-Iran, AB720068-Iran, AB720066-Iran, KX186705-Iran, AB720069-Iran, KT318130-Iran, KX186704-Iran, KX186699-Iran, KT033489-Iran, KT318129-Iran, AJ132907-Poland, AJ132908-Poland, AJ132909-Poland, AJ132910-Poland, MH986751-Poland, JX266825-Poland, MN444805-Poland, JX266826-Poland, MH986750-Poland, MH986749-Poland, HAJ237639-Poland, MN444804-Poland, JF751034-Canada, KC848475-Canada, KC848476-Canada, KC848477-Canada, KF962559-Canada, KF962566-Canada, KF962567-Canada, KF962568-Canada, OK095088-Canada, KX384668-Sweden, KX384669-Sweden, KX384670-Sweden, KX384671-Sweden, KY094609-China, EU704123-China, KU723572-China, MH259775-China, AY389984-China, EU704122-China, EU704124-China, MH259776-China, MH259777-China, MH259778-China, MK248696-Turkey, MK248702-Turkey, MK248703-Turkey, MK248704-Turkey, MK248705-Turkey, MK248706-Turkey, MW326786-Slovakia, MW326787-Slovakia, MN444803-Slovakia, MW343787-Slovakia, MW343788-Slovakia, MN444802-Slovakia, MN444801-Slovakia, MW343789-Slovakia, MW357715-Slovakia, MW366778-Slovakia, MW366779-Slovakia, MW384819-Slovakia, MW384820-Slovakia, AB668376-Germany, MN251882-Austria, MN444806-Austria, MN444799-Hungary, MN444800-Hungary, KR870967-Switzerland, AY855918-Estonia, MW560731-Slovenia, OM640356-Kazakhstan
Hap02	16	KC848462-Canada, KC848464-Canada, KC848465-Canada, KC848466-Canada, KC848467-Canada, KC848468-Canada, KC848469-Canada, KC848470-Canada, KC848471-Canada, KC848472-Canada, KC848473-Canada, KF962555-Canada, KF962562-Canada, KF962563-Canada, KF962564-Canada, KF962565-Canada
Hap03	1	KF962556-Canada
Hap04	1	KF962557-Canada
Hap05	1	KF962558-Canada
Hap06	1	KF962560-Canada
Hap07	1	KF962561-Canada
Hap08	1	KF962569-Canada
Hap09	1	KF962570-Canada
Hap10	1	KF962571-Canada
Hap11	1	MN448475-China
Hap12	1	MN448476-China
Hap13	1	AJ237640-Germany

### Analyses of neutrality, diversity, and gene flow

Table 4 depicts the values of neutrality and diversity indices for both genes, i.e., mt-*cox*1 and mt-*nad*1. Tajima’s D and Fu’s FS were computed to determine the selection pressure on the population of the parasite. The presence of a higher number of alleles was depicted through negative values in the mt-*cox*1 and mt-*nad*1 areas obtained from Tajima D and Fu’s FS.

## Discussion

Animals throughout the world are vulnerable to parasite-borne acute, chronic, and severe illnesses [23–25]. Parasitic diseases are responsible for huge economic losses in terms of loss of production and medicinal costs [26,27]. In modern-day science, where parasites are believed to have evolved according to their respective habitat and ecosystem, it is becoming important to have knowledge about the genetic diversity of the parasites for a better understanding of the transmission dynamics of parasites so that effective monitoring and control measures may be devised [28,29].

Population structure and genetic diversity of *E. multiloclaris* were examined during the study using two specialized genes, i.e., mt-*cox*1 and mt-*nad*1, which are frequently used to distinguish among the species of *Echinococcus* parasite. The nucleotide sequences of these genes were downloaded from GenBank. The present study revealed the global epidemicity of infections caused by *Echinococcus,* along with the genetic diversity, population structure, and gene flow of the parasite. For this purpose, a total of 275 mt-*cox*1 genes (497 bp) and 124 mt-*nad*1 (285 bp) gene sequences that have already been registered in the NCBI database were used.

The major factor behind the haplotype variation between different researchers is the length of the gene sequence selected for the study of the parasite. The longer the length of the genes selected for genetic studies, the greater the number of haplotypes identified upon mt gene sequencing. Out of a total of 399 nucleotide sequences, 20 haplotypes of mt-*cox*1 gene and 13 mt-*nad*1 genes were examined. A similar study has been conducted by Kinkar et al. [30] that revealed high genetic diversity among *E. granulosus*. The scientist has identified 171 haplotypes of* E. granulosus* among 212 samples (haplotype diversity = 0.994), which shows almost the entire mt sequence [30].

**Table 4.** Diversity and neutrality indices were obtained using nucleotide data of the mt-*cox*1 (497 bp) and mt-*nad*1 (285 bp) genes of *E*. *multilocularis*.

Indices	*nad*1 (285 bp)	*cox*1 (497 bp)
No. of sequences	124	275
No. of mutations	14	24
Parsimony informative sites	3	1
No. of haplotypes	13	20
Haplotype diversity (Hd)	0.374 ± 0.052	0.140 ± 0.029
Nucleotide diversity (π)	0.00287 ± 0.00045	0.00111 ± 0.00026
Tajima’s *D *	−1.89245	−2.53266
Fu’s Fs	−9.568	−41.675
FLD	−4.94806	−9.15087
FLF	−4.56645	−7.75910

Neutrality indices were computed to evaluate population growth and nucleotide variability [31]. The Tajima’s D model measures divergence in populations from the conventional neutral model. Where a positive value indicates heterozygosity (having selected advantage), while the negative Tajima’s D value indicates that one allele has a selective advantage over the other allele and significant growth in population [32]. In the present study, the values of Tajima’s D were found to be very low for both of the genes, i.e., mt-*cox*1 and mt-*nad*1, which indicate a maximum likelihood for future population expansion. The value of Tajima’s D for mt-*nad*1 gene was lower (−1.89245) than that of mt-*cox*1 (−2.53266), which depicts the rapid increase in the population of the former gene. The negative values of neutrality indices indicate migration across different countries, which, according to Tajima’s D, indicates more population expansion in the coming years.

Another marker for population growth sensitivity is Fu’s FS. Through this method, we can identify whether the gene pool of populations of different parasite species is the same and reveal identical tendencies in growth or not [33,34]. The values of Fu’s FS for both mt-*cox*1 and mt-*nad*1 genes were found to be extremely low, which shows that worldwide expansion in the population of parasites could be expected.

Nucleotide diversity was used to assess the polymorphism in the population. The mean nucleotide difference of mt-*nad*1 gene (0.00287) was found to be greater than that of mt-*cox*1 gene (0.00111). Haplotype diversity was also calculated to identify the uniqueness of the haplotypes within the populations. In the present investigation, marginal differences were identified in the gene sequences of mt-*nad*1 gene (0.374) and mt-*cox*1 gene (0.140). A total of 20 haplotypes were discovered in the mt-*cox*1 gene. Out of these, 18 haplotypes were identified as distinct haplotypes, accounting for 92.72% of the network. The examination of mt-*nad*1 gene sequences revealed 13 distinct haplotypes having 78.82% as the dominant primary haplotype of the network. Furthermore, major haplotypes represented a single ancestor. Mutation rates of both genes, i.e., mt-cox1 and mt-*nad*1 were analyzed, which revealed 24 mutations in mt-*cox*1 (497 bp) and 14 mutations in mt-*nad*1 gene (285 bp). The higher mutation rates in both genes indicate the extensive and complicated evolutionary history of *E. multilocularis*. Extensive diversity in the genetic makeup of *E. multilocularis* has been reported worldwide. Furthermore, the complex phylogenetic associations identified through geographic and phylogenetic analyses highlighted the significant role of animal trade in the present distribution of *E. granulosus* [30].

Due to a lack of investigations into the population structure of *E*.* multilocularis*, an extensive comparative study was conducted by comparing the results of this study with those reported by other scientists worldwide. However, it is a fact that the length of the selected gene sequences has influenced the genetic variation of the parasite, e.g., *E. granulosus* [35,36]. It is further supported by a study that selected 223 European isolates and 89 Italian isolates from the same gene, mt-*cox*1. The higher number of isolates revealed 24 haplotypes, while the lower number of isolates revealed seven haplotypes [37]. Similarly, seven haplotypes were discovered in 69 Argentinian isolates of *cox*1 gene [38]. Another study, which was conducted in the Sindh province of Pakistan, revealed five haplotypes out of 112 isolates of *cox*1 gene sequence [39]. Furthermore, findings from another Pakistani study that included the full-length *cox*1 and *nad*1 genes were much more illustrative than those studying fragmentary sequences [40]. The entire sequence may provide more effective evidence to verify the validity of *E. granulosus* genotypes. Based on these observations, it is recommended that future studies about *E. multilocularis* employ full-length gene amplification rather than using a partial segment of the genes.

## Conclusion

Despite the fact that multiple molecular investigations have been carried out, the present study is the maiden attempt to investigate the population structure of *E. multilocularis*. Low diversity between the selected genes of *E. multilocularis*, i.e., mt-*cox*1 and mt-*nad*1 was found during the present study. For the whole population, the values of Tajima’s D and Fu’s FS were found to be negative, though statistically insignificant. The findings of the study will add to the existing bank of knowledge about the genetic diversity of *E. multilocularis* and also provide baseline data for future epidemiological insights into the population structure of *E. multilocularis*.

## List of Abbreviations

AE, Alveolar echinococcosis; *cox*1, cytochrome c oxidase I; *nad*1, NADH dehydrogenase; NCBI, National Center for Biotechnology Information; Hap, Haplotype; MEGA, Molecular Evolutionary Genetics Analysis Software; MCMC, Markov Chain Monte Carlo; mt, Mitochondrial; NCBI, National Center for Biotechnology Information
